# A Case of Chronic Conjunctivitis following Rituximab Therapy

**DOI:** 10.1155/2009/272495

**Published:** 2009-09-01

**Authors:** Marnelli A. Bautista, Walter D. Y. Quan, Jun Wang

**Affiliations:** ^1^Department of Pathology and Laboratory Medicine, Loma Linda University Medical Center, 11234 Anderson Street, Loma Linda, CA 92354, USA; ^2^Division of Medical Oncology, Department of Internal Medicine, Loma Linda University School of Medicine, 11175 Campus Street, Chan Shun Pavilion 11015, Loma Linda, CA 92354, USA

## Abstract

The activity of the anti-CD20 monoclonal antibody, rituximab in B-cell non-Hodgkin's lymphoma, with relatively minimal toxicity has been well established. Adverse effects such as low-grade fever, urticaria, bronchospasm, sporadic tachycardia, and hypotension have been described. However, only a single case of rituximab-related, transient conjunctivitis has been documented in literature. We report an occurrence of chronic, bilateral conjunctivitis in an 88-year-old female diagnosed with stage IV, non-Hodgkin's lymphoma (NHL), who was maintained on rituximab for 12 months. In contrast to the previously described case, our patient developed severe conjunctival inflammation approximately three to four weeks following rituximab induction. Resolution of conjunctivitis occurred within two months after cessation of rituximab treatment.

## 1. Introduction

The chimeric, anti-CD20 monoclonal antibody, rituximab plays a significant role in the management of B-cell non-Hodgkin's lymphomas. Benefit from this agent has been observed in both low-grade and high-grade lesions [1–5]. In addition, its low-toxicity profile has made it a suitable preference as part of the initial treatment panel for most patients with B-cell lymphoma. Adverse reactions consist of low-grade fever, urticaria, rhinitis, lacrimation, bronchospasm, occasional hypotension, and tachycardia. Only one case of conjunctivitis has been reported [6]. 

We report a case of significant, chronic conjunctivitis in an 88-year-old female with low-grade follicular lymphoma, who was maintained on rituximab for one year. The patient developed mild, bilateral eye inflammation approximately three to four weeks within the induction cycle. She experienced aggravation of the conjunctivitis with subsequent rituximab treatments.

## 2. Case Report

An 88-year-old female with insidious onset of dyspnea on exertion and occasional nonproductive cough was found to have a 5.7 cm right hilar mass on chest x-ray. A CT-guided biopsy of the soft tissue mass showed a diffuse, monotonous infiltrate of small to medium-sized lymphocytes. Mitotic figures were not significantly increased, with only approximately 5% of the tumor cell nuclei exhibiting Ki-67 reactivity. The predominant lymphocytes were positive for CD20, CD10, and Bcl-6 and negative for CD3 and Cyclin D1 markers. 

**Figure 1:**
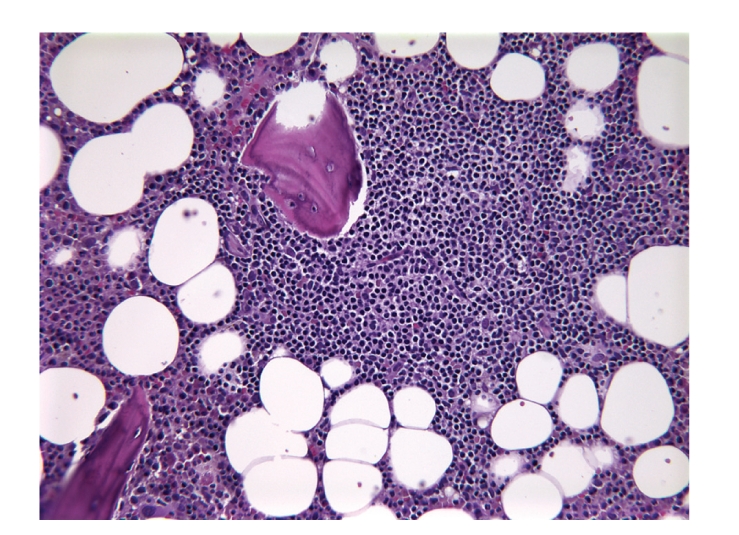
Bone marrow trephine core biopsy shows a single paratrabecular lymphoid aggregate, characteristic of bone marrow involvement by follicular lymphoma (hematoxylin and eosin stain, 20x).

Bone marrow biopsy was performed to assess systemic involvement. The core biopsy revealed a normocellular marrow for age with a single, paratrabecular lymphoid aggregate (Figure 1), demonstrating similar immunohistochemical staining pattern to that of the right hilar mass. The characteristic features of the malignant cells in the mediastinum and bone marrow were consistent with a stage IV, low-grade, follicular B-cell lymphoma. 

Radiation to the mediastinal mass was initiated with concomitant rituximab treatment (375 mg/m^2^ for four weeks). No adverse reactions were noted during the first two weeks of rituximab induction. However, the patient developed mild, bilateral ocular pruritis and lacrimation three to four weeks within the induction cycle. Maintenance treatment with rituximab (four weekly doses of 375 mg/m^2^ every six months) was pursued since the patient tolerated the induction cycle without severe complications. Nevertheless, the bilateral eye inflammation became more pronounced after the second cycle of maintenance therapy. In addition to the pruritis and lacrimation, she also developed intense, bilateral periocular erythema, moderate edema, and pain as well as gradual blurring of vision. These manifestations were more prominent in the left eye. She had neither new lymphadenopathy nor any other systemic symptoms. She was then referred to an ophthalmologist for abatement of symptoms and was given topical steroid drops.

**Figure 2:**
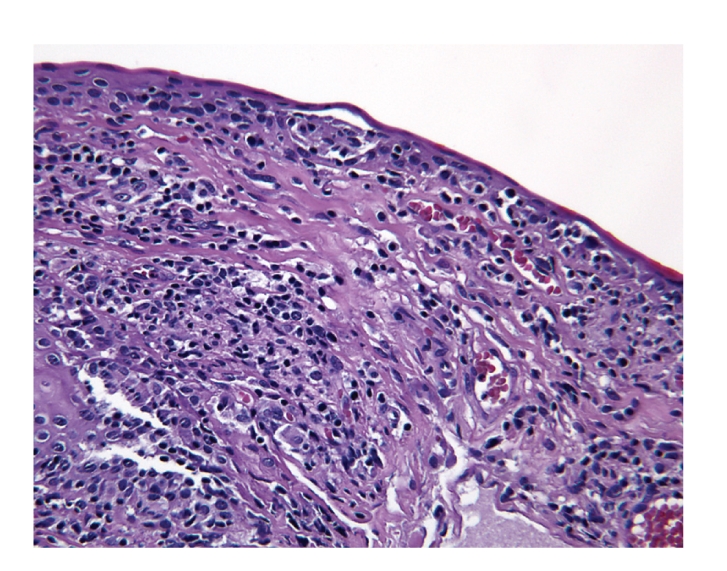
Left conjunctival biopsy with diffuse lymphocytic infiltrate (hematoxylin and eosin stain, 20x).

**Figure 3:**
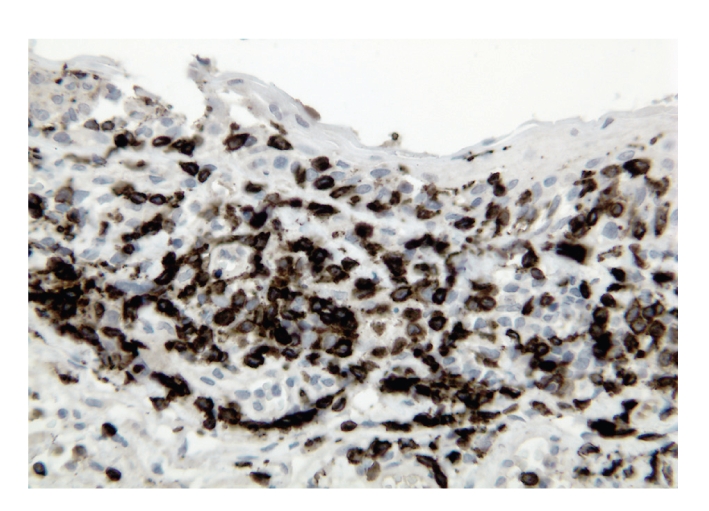
The vast majority of the lymphoid cells in the left conjunctival biopsy are CD3-positive T lymphocytes (immunoperoxidase staining, 40x).

**Figure 4:**
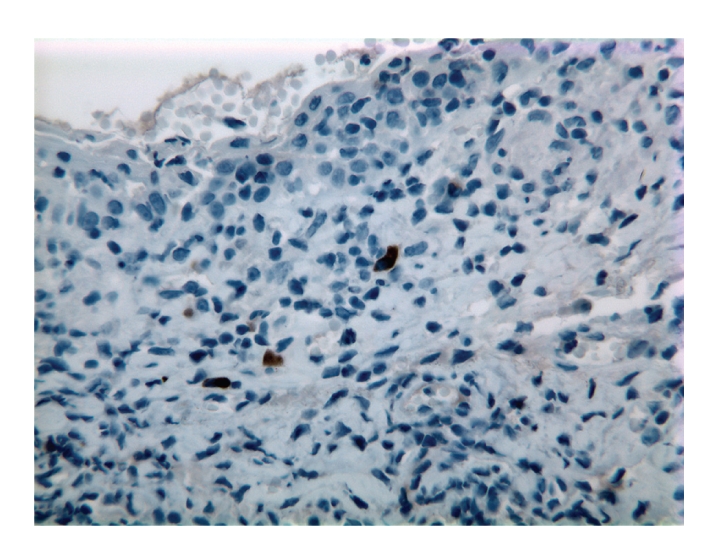
Rare B lymphocytes are positive for CD79a (left conjunctiva, immunoperoxidase staining, 40x).

**Figure 5:**
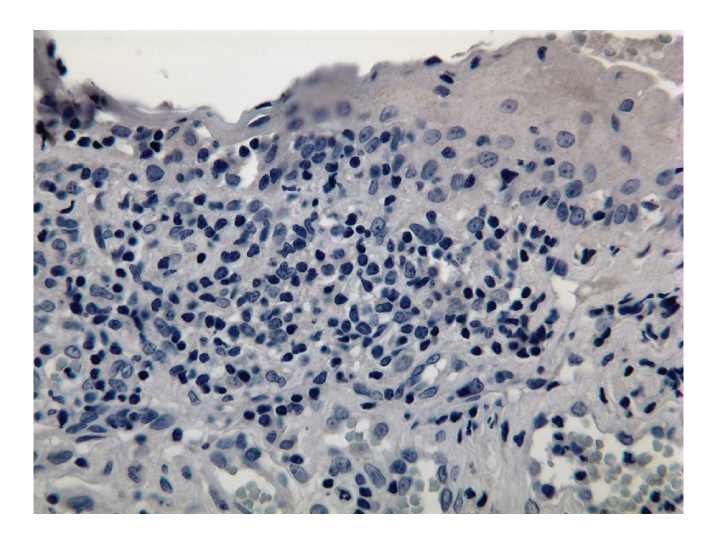
The lymphocytes are negative for CD20 (left conjunctiva, immunoperoxidase staining, 40x).

Concurrent CT of the chest, abdomen, pelvis, and bilateral orbits revealed no evidence of malignancy or disease recurrence. A left eye conjunctival biopsy was performed to further exclude the possibility of an underlying neoplasm. The biopsy showed an intact epithelium, prominent lymphatic channels with increased stromal lymphocytes (Figure 2). The majority of the lymphocytes expressed CD3, a T-cell marker (Figure 3), with only rare cells reactive for CD79a, a B-cell marker (Figure 4). The CD20 stain was negative (Figure 5). 

The severe ocular symptoms gradually subsided within approximately four to six weeks after the last dose of the second rituximab maintenance cycle. However, mild inflammation of the left eye persisted. Moreover, exacerbation of the left eye conjunctivitis was noted one week after the patient received her initial rituximab dose on the third cycle of maintenance regimen. Rituximab was discontinued, and a followup ophthalmology visit was made. Topical antibiotic and steroid drops were utilized for approximately seven days. Two to three weeks after the termination of rituximab, the patient's conjunctivitis has markedly subsided and has completely resolved in the succeeding eight weeks. 

## 3. Discussion

The efficacy of rituximab as a combination therapy or a single, maintenance regimen has been well documented in the treatment of non-Hodgkin's lymphoma, including the follicular subtype [1–5]. Recent evidence that the use of maintenance rituximab significantly prolongs disease-free survival in patients with follicular histology, and with minimal toxicity, further suggests that more patients may be treated with this agent in the future [7]. Indeed, this monoclonal antibody has been referred to as “the most important advance in the treatment of B-cell lymphoma in the past 30 years” [8]. 

Toxicity related to this therapy includes fever, chills, urticaria, bronchospasm, hypotension, elevated heart rate, and infrequently, rhinitis, and lacrimation [4, 5]. Only one case of transient conjunctivitis associated with rituximab infusion has been reported in literature, thus far [6]. However, in contrast to the aforementioned case, our patient developed a delayed-type, hypersensitivity reaction, manifesting as persistent, chronic conjunctivitis.

Conjunctival inflammation can be triggered by irritative substances, allergens, or underlying viral or bacterial infections. In particular, immunocompromised or immunosuppressed states as a consequence of immune incapacitating disease processes or the use of medications, such as immunosuppressants or chemotherapy, increase the risk of acquiring infections [9], and hence, conjunctivitis. In addition, chemotherapy agents themselves, such as cytosine arabinoside, may be irritating to the conjunctiva based on the drug concentration present in tears [10]. 

In order to exclude a neoplastic process such as metastatic involvement of the conjunctiva, a biopsy with immunohistochemical analysis was accomplished. We have pursued an auxiliary B-cell marker investigation such as immunohistochemical staining for CD79a, in addition to CD20 to evaluate the presence of malignant B lymphocytes that may have lost surface expression of CD20 after treatment with rituximab [11]. Predominance of CD3 reactive T lymphocytes with only rare, scattered B lymphocytes positive for CD79a further substantiated a reactive process.

The exact pathophysiologic mechanism as to how rituximab triggers conjunctivitis is not yet clearly elucidated, but it can possibly be attributed to complement activation with release of cytokines [12]. Myelosuppression and decline in selected immunoglobulin levels and humoral function, as a result of rituximab's anti-CD20 effect [4, 12, 13], may also impair immune response. As such, conjunctival inflammation could be prompted or aggravated by concomitant, opportunistic infections.

## 4. Conclusion

Our case emphasizes a rare but clinically significant occurrence of rituximab-associated chronic conjunctivitis. Awareness of this particular adverse reaction is needed by the prudent clinician, primarily as the role of rituximab expands.

**Consent**

Written informed consent was obtained from the patient for publication of this case report and accompanying images. A copy of the written consent is available for review by the Editor-in-Chief of this journal.

## Abbreviations

CD: Cluster of differentiation
CT: Computed tomography
NHL: Non-Hodgkin's lymphoma.
